# Rifampin-Resistant *Mycobacterium bovis* BCG–Induced Disease in HIV-Infected Infant, Vietnam

**DOI:** 10.3201/eid1907.130025

**Published:** 2013-07

**Authors:** Duc Nguyen Hong, Mai Nguyet Thu Huyen, Nguyen Thi Ngoc Lan, Nguyen Huy Duong, Vi Vi Nguyen Ngo, Duong Tran Ngoc, Khanh Truong Huu, Tuyen Nguyen, Viet Do Chau, Oliver Marcy, Philippe Van de Perre, Anne-Laure Bañuls, Sylvain Godreuil

**Affiliations:** Pham Ngoc Thach Hospital, Ho Chi Minh City, Vietnam (D. Nguyen Hong, M.N.T. Huyen, N.T.N. Lan, N.H. Duong, V.V. Nguyen Ngo, D.T. Ngoc, T Nguyen);; Pediatric Hospital Nhi Dong 1, Ho Chi Minh City (K.T. Huu);; Pediatric Hospital Nhi Dong 2, Ho Chi Minh City (V.D. Chau);; Institut Pasteur, Phnom Penh, Cambodia (O. Marcy);; INSERM U 1058, Montpellier, France (P. Van de Perre, S. Godreuil);; Université Montpellier 1, Montpellier (P. Van de Perre, S. Godreuil);; CHU Montpellier, Montpellier (P. Van de Perre, S. Godreuil);; MIVEGEC, Universités Montpellier 1 et 2, Montpellier (A.-L. Bañuls)

**Keywords:** Mycobacterium bovis Bacillus Calmette-Guérin, rifampin resistance, BCG disease, HIV-infected child, Vietnam, tuberculosis and other mycobacteria, antimicrobial resistance, bacteria, HIV, vaccines

**To the Editor:** Guidelines for the diagnosis and management of *Mycobacterium bovis* BCG disease in HIV-infected children are lacking. BCG strains are intrinsically resistant to pyrazinamide and in some cases have low-level resistance to isoniazid (*6*). However, data on acquired drug resistance in *M. bovis* BCG are limited. We describe a case of BCG disease caused by a rifampin-resistant strain of *M. bovis* BCG in an HIV-infected infant in Vietnam.

The daughter of a known HIV-infected woman, who did not fully adhere to antiretroviral therapy (ART) during pregnancy, received the *M. bovis* intradermal BCG (Pasteur strain) vaccine at birth. HIV infection was diagnosed in the infant by PCR when she was 8 weeks of age. At 9 months of age, she was admitted to the Pediatric Infectious Diseases Department of the Pham Ngoc Thach Hospital (Ho Chi Minh City, Vietnam) because of a voluminous ipsilateral axillary mass at the site of the vaccination, fever, weight loss, and hepatosplenomegaly. The percentage of CD4+ T cells was 27% (1,620 cells/mm^3^). Regional BCG disease was clinically diagnosed without microbiological investigation, and a broad antimycobacterial therapy targeting *M. tuberculosis* complex species was started with 5 mg/kg isoniazid, 10 mg/kg rifampin, and 25 mg/kg pyrazinamide. After 6 weeks of antimycobacterial therapy, ART was initiated with lamivudine, stavudine, and abacavir. 

After 6 months of antimycobacterial treatment, the infant was hospitalized again for recurrent inflammation and fistulization of the axillary lymph nodes associated with fever. Fluid from the axillary mass was collected by fine-needle aspiration for bacteriologic investigations. Direct microscopic examination showed acid-fast bacilli, and the mycobacterial infection was confirmed by culture. By using conventional biochemical methods, the mycobacterial isolate was assigned to the *M. bovis* species. Pyrazinamide was discontinued, and antimycobacterial therapy was continued for 4 supplementary months with rifampin (15 mg/kg) and isoniazid (10 mg/kg). After 2 months, drug susceptibility testing results confirmed pyrazinamide intrinsic resistance and isoniazid and ethambutol susceptibility and showed rifampin resistance. The late inflammatory reaction after introduction of ART was evocative of immune reconstitution inflammatory syndrome. Nevertheless, drug resistance may have contributed. Despite the rifampin resistance, the patient showed clinical improvement, and the rifampin/isoniazid treatment was continued for 2 more months. The child’s BCG disease was cured on completion of 10 months of antituberculous treatment.

Retrospective molecular investigations using the GenoType MTBC Kit (Hain Lifescience, Nehren, Germany) enabled identification of the isolate stored at −80°C as *M. bovis* BCG strain. A mutation in the *rpoB* gene (codon 531, Ser531Tyr) associated with rifampin resistance was detected by using the GenoType MTBDRplus Kit (Hain Lifescience) and partial sequencing of the *rpoB* gene (*5*,*7*). No mutation in the *katG* and *inhA* genes, frequently associated with isoniazid resistance, was detected.

To our knowledge, this case is the second report of rifampin-resistant *M. bovis* BCG disease in HIV-infected children. The first report involved a child in South Africa who was vaccinated with the Danish BCG strain (*4*); this strain shows low-level resistance to isoniazid and therefore has a high risk of evolving to multidrug resistance in instances of suboptimal isoniazid levels. The *M. bovis* BCG Pasteur strain (American Type Culture Collection 35734) used for vaccination in Vietnam is isoniazid and rifampin susceptible and pyrazinamide resistant (*9*). Despite appropriate antimycobacterial treatment, the relatively low doses of isoniazid (5 mg/kg), poor adherence, or inadequate absorption of drugs because of HIV-related gastrointestinal disease may have resulted in subtherapeutic in vivo drug concentrations and thus in selection of a drug-resistant *M. bovis* BCG strain. This case should alert clinicians of the possible emergence of rifampin resistant *M. bovis* BCG strains.

Because disseminated BCG disease in HIV-infected children presents a high risk for illness and/or death, these patients should receive optimal tuberculosis treatment (*2*) based on 4-drug (rifampin, isoniazid, ethambutol, and pyrazinamide) regimen doses for at least 9 months until *M. tuberculosis* is ruled out (*3*). Untreated local BCG immune reconstitution inflammatory syndrome may not necessarily progress to dissemination; therefore, treatment would not appear necessary (*8*).

Some studies suggest that the survival of HIV-infected children with BCG disease could be attributed to early initiation of ART in combination with other treatments (*1*,*3*). In the South Africa case, the child died, and the authors suggested that this outcome was related to the severity of the clinical features, the severe HIV-related immune suppression, and the absence of ART (*4*). In the case in Vietnam, despite the emergence of drug resistance, the early initiation of ART in a child with a localized disease, the persistent efficacy of isoniazid, and the spontaneous fistulization of the abscess probably contributed to the good outcome for the infant.

In conclusion, this case highlights the challenges in management of BCG disease in children. It also emphasizes the possible risk for emergence of acquired drug resistance in *M. bovis* BCG strains, complicating the medical management of such cases.
